# iT4SE-EP: Accurate Identification of Bacterial Type IV Secreted Effectors by Exploring Evolutionary Features from Two PSI-BLAST Profiles

**DOI:** 10.3390/molecules26092487

**Published:** 2021-04-24

**Authors:** Haitao Han, Chenchen Ding, Xin Cheng, Xiuzhi Sang, Taigang Liu

**Affiliations:** College of Information Technology, Shanghai Ocean University, Shanghai 201306, China; m190711268@st.shou.edu.cn (H.H.); m190711300@st.shou.edu.cn (C.D.); m200701415@st.shou.edu.cn (X.C.); m180711086@st.shou.edu.cn (X.S.)

**Keywords:** type IV secreted effectors, support vector machine, random forest, position-specific scoring matrix, position-specific frequency matrix

## Abstract

Many gram-negative bacteria use type IV secretion systems to deliver effector molecules to a wide range of target cells. These substrate proteins, which are called type IV secreted effectors (T4SE), manipulate host cell processes during infection, often resulting in severe diseases or even death of the host. Therefore, identification of putative T4SEs has become a very active research topic in bioinformatics due to its vital roles in understanding host-pathogen interactions. PSI-BLAST profiles have been experimentally validated to provide important and discriminatory evolutionary information for various protein classification tasks. In the present study, an accurate computational predictor termed iT4SE-EP was developed for identifying T4SEs by extracting evolutionary features from the position-specific scoring matrix and the position-specific frequency matrix profiles. First, four types of encoding strategies were designed to transform protein sequences into fixed-length feature vectors based on the two profiles. Then, the feature selection technique based on the random forest algorithm was utilized to reduce redundant or irrelevant features without much loss of information. Finally, the optimal features were input into a support vector machine classifier to carry out the prediction of T4SEs. Our experimental results demonstrated that iT4SE-EP outperformed most of existing methods based on the independent dataset test.

## 1. Introduction

Gram-negative bacteria use various secretion systems to secrete their virulence factors (mainly of proteins) to invade the host cells. Until now, the secretion systems of gram-negative bacteria are divided into eight categories (I–VIII) according to their outer membrane secretion mechanisms [1]. Among them, the type IV secretion systems (T4SS) are specialized ATP-dependent protein complexes which are used by a variety of bacterial pathogens to deliver effector molecules into the cytosol of eukaryotic host cells [2]. These transported proteins, called type IV secreted effectors (T4SE), can manipulate host cell gene expression and processes, exploit the host cell machinery for their own profit, and escape the immune responses during infection, often resulting in severe diseases or even death of the host [3]. Therefore, identification of putative T4SEs is the first important step towards understanding host-pathogen interactions and bacterial pathogenesis. However, it is often quite time-consuming and laborious to identify new T4SEs based on wet-lab experimental approaches, such as fusion protein report assays and secretion apparatus [4]. In many cases T4SEs contain a wide range of signal characteristics including eukaryotic-like domains, localization signals, and C-terminal translocation features [5], which enables bioinformatics-based methods to predict T4SEs more efficiently through extracting features as inputs of machine learning models.

In recent years, various computational tools based on machine learning and deep learning have been developed to identify T4SEs using protein sequences information, including T4EffPred [6], T4SEpre [4], PredT4SE-Stack [7], DeepT4 [8], Bastion4 [9], OPT4e [10], CNN-T4SE [11], T4SE-XGB [12], and so on [13,14,15]. For instance, Wang et al. [4] developed an effective inter-species T4SEs prediction software package, termed T4SEpre, which first carefully compared C-terminal sequential and position-specific amino acid composition (AAC), possible motifs and structural features and then fused these features to train a support vector machine (SVM) model. Based on the 5-fold cross validation (CV), T4SEpre could distinguish the T4SEs from the non-T4SEs with the sensitivity of 89% and the specificity of 97% [4]. In order to verify the contribution of the N-terminal sequence features to the identification of T4SEs, Wang et al. [15] first established a benchmark dataset which consists of 380 T4SEs and 1,385 non-T4SEs, and then made systematical comparisons of the N-terminal and C-terminal regions. Finally, they proposed an accurate SVM-based model for the annotation of T4SEs by utilizing three different types of sequence information, namely AAC, composition, transition, and distribution (CTD), and position-specific scoring matrix (PSSM). Based on the same datasets, Xiong et al. [7] explored a stacked ensemble model, namely PredT4SE-Stack, to predict whether a query protein is a T4SE or not based on its PSSM-composition features, which employs an ensemble of base-learners, such as SVM, gradient boosting machine (GBM), and extremely randomized trees (ERT), to generate outputs of the meta-classifier. The experimental results on the CV and the independent tests indicated that PredT4SE-Stack model further improved the prediction performance of T4SEs with the help of two stages of learning [7]. Recently, Wang et al. [9] comprehensively assessed the performance of different protein encoding schemes and their combinations along with several machine learning algorithms for T4SEs identification based on their self-constructed working datasets. Their study showed that PSSM-based model achieved the best prediction performance for all classifiers and ensemble model performed better than these individual single-feature models [9]. In addition, they developed an online web server, called Bastion4, to facilitate the prediction of T4SEs [9]. Over the past decade, deep learning approaches have been widely used in bioinformatics and other related fields [16,17]. Hong et al. [11] applied a convolution neural network (CNN) technique to annotate T4SEs by integrating three protein encoding strategies, i.e., PSSM, protein secondary structure and solvent accessibility (PSSSA), and one-hot encoding scheme (Onehot). They also constructed a novel annotation tool CNN-T4SE to provide the online prediction of T4SEs with improved accuracy and reduced false positive rate [11]. Although deep learning has achieved remarkable success in various fields, it needs a large amount of data as input to help train a better model. However, due to a limited number of known T4SEs, the generalization and prediction ability of deep learning models need to be enhanced.

For these traditional machine learning models, the design of a high-quality protein encoding strategy is a crucial step towards improving the recognition ability of T4SEs based on sequence data. Previous studies have demonstrated that evolutionary information encoded in the PSSM is more informative than sequence itself and various PSSM-based feature descriptors have been successfully applied to enhance the identification performance of T4SEs and other protein attributes [7,9,18]. In general, the PSSM of a query protein is an *L* × 20 matrix calculated by using the PSI-BLAST software package [19] to iteratively search a given protein against a specified database to detect its distantly related homologous proteins above a specified e-value score, where *L* is the length of the query sequence and the columns of the matrix represent 20 natural amino acids. Actually, the return profiles of the PSI-BLAST program consist of two *L* × 20 matrices. The first one is called PSSM and gives the log odds of the substitution score for each amino acid. The second one is called position-specific frequency matrix (PSFM) which displays the weighted observed percentages of amino acid substitutions. To our knowledge, PSFM is rarely used to generate sequence descriptors for the classification of proteins. Hence, the evolutionary information in the form of PSI-BLAST profiles has not been adequately explored for the annotation of T4SEs in earlier studies.

**Figure 1:**
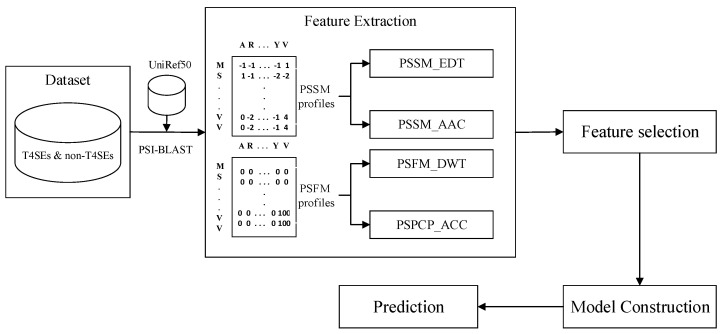
System diagram of the proposed method.

In this work, we proposed a novel machine learning model named iT4SE-EP to further improve the accuracy of the T4SEs prediction by extracting more informative features from two PSI-BLAST profiles. The workflow diagram of the iT4SE-EP method is illustrated in Figure 1. First, the PSSM and PSFM profiles of query proteins were transformed into fixed-length feature vectors by using four types of encoding techniques, i.e., AAC, auto-covariance and cross-covariance (ACC), evolutionary difference transformation (EDT), and discrete wavelet transform (DWT). Then, the random forest (RF) algorithm was applied to rank these hybrid features according to their classification ability. Finally, the optimal feature subsets were selected as the input of an SVM classifier for the computational identification of T4SEs. To comprehensively evaluate the performance of the proposed iT4SE-EP model, the jackknife CV and the independent test were performed on two widely used benchmark datasets. The experimental results demonstrated that iT4SE-EP is very promising and could be used to help increase annotation levels of T4SEs.

## 2. Results and Discussions

### 2.1. The Effect of Parameter D on the Prediction Performance

**Figure 2:**
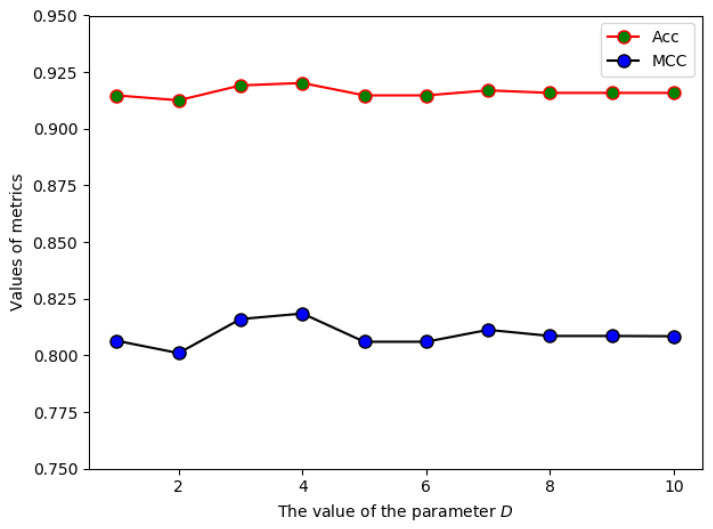
The performance of PSSM_EDT with different *D* on the Train-915 dataset.

In the PSSM_EDT model, the value of parameter D would affect the dimension of the feature vector and the performance of the predictor (see Materials and Methods section). D can be any integer between 1 and L−1, where *L* is the shortest length of sequences in the training dataset. Since too large D could cause the curse of dimensionality, we set the maximum value of D to 10. The value of D was optimized on the Train-915 dataset by using the SVM classifier and the jackknife test. The accuracy (Acc) and Matthew’s correlation coefficient (MCC) were selected as the evaluation measures. The performance of the PSSM_EDT model with different values of D was shown in Figure 2, from which we can see that the best Acc and MCC metrics were achieved when D=4. Therefore, the optimal size of the PSSM_EDT descriptor was 400 × D=1600 for the further study.

### 2.2. Comparative Analysis of Different Classifiers with Different Feature Encoding Schemes

**Figure 3:**
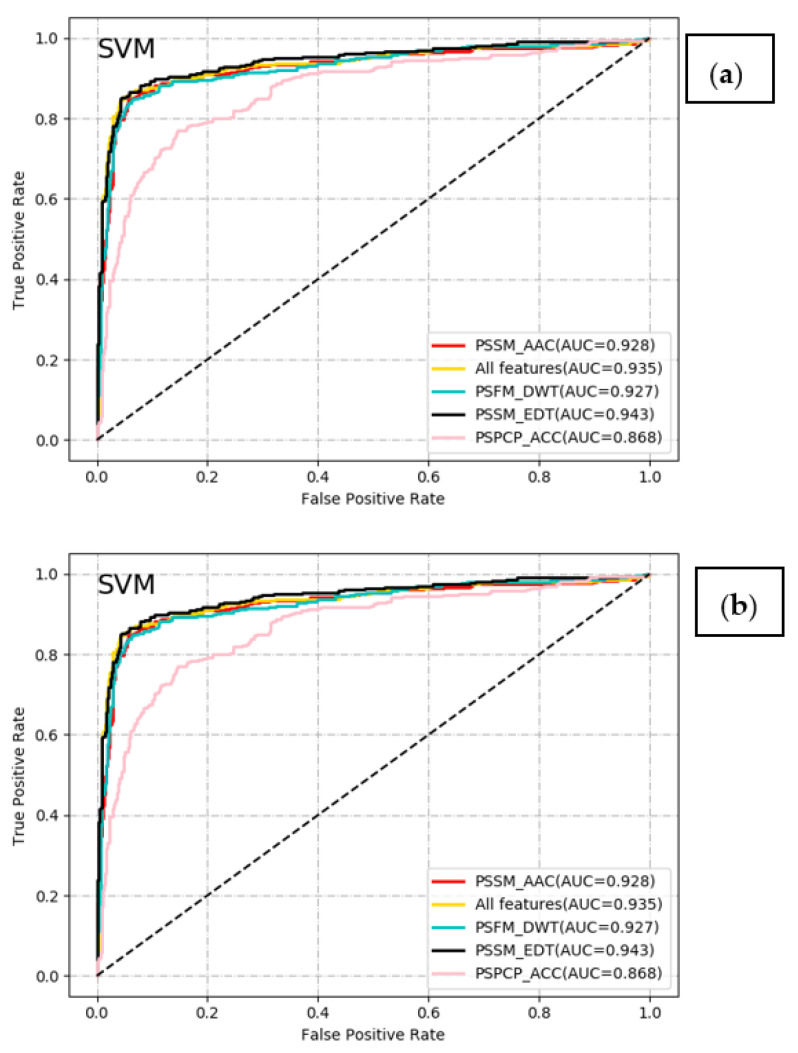
ROC curves of the SVM and RF predictors with different features. (**a**) ROC curves based on the SVM predictors. (**b**) ROC curves based on the RF predictors.

In this section, we compared the performance between the SVM and RF classifiers combined with each of the five different feature encoding schemes. Results on the Train-915 dataset by using the jackknife CV were shown in Table 1 and Figure 3.

As can be seen from Table 1, the SVM models based on PSSM_AAC, PSFM_DWT, PSSM_EDT, and all the fused features exhibited satisfying performance with the Acc and the AUC higher than 0.9, while the SVM model with PSPCP_ACC obtained the acceptable Acc value (0.834) and AUC value (0.868). This indicated that the proposed four types of feature encoding methods could provide some valuable clues for the identification of T4SEs. In addition, the combination of SVM and PSSM_EDT was superior to other models in terms of Acc (0.920), MCC (0.818), F-score (0.876), and Pre (0.908). Meanwhile, the SVM models based on PSSM_EDT and all features achieved the highest Sen, while the ones based on PSSM_EDT and PSSM_DWT obtained the best Spe, indicating that PSSM_EDT and PSSM_DWT features may be crucial to the prediction of T4SEs. However, the performance of the SVM model with all features was slightly poorer than that of the SVM model with only PSSM_EDT features, which suggests that irrelevant and redundant features existed in the four types of features have an unfavorable impact on the performance of the model. For the RF classifier, the model based on all features outperformed other models with the single-view feature in terms of Acc (0.907), MCC (0.788), Sen (0.816), F-score (0.854), and Pre (0.895). Obviously, the SVM-based models showed the better performance than the RF-based models in this study. Similar conclusions were illustrated in Figure 3. Moreover, the Sen values obtained by these models were significantly lower than the Spe values. This may be caused by the unbalanced sample sizes between T4SEs and non-T4SEs in the datasets. To balance the Sen and the Spe, we performed the models again by adjusting the default probability cut-off from 0.5 to 0.4. The corresponding results were seen in Supplementary Table S1.

### 2.3. Performance Analysis of Feature Selection

**Figure 4:**
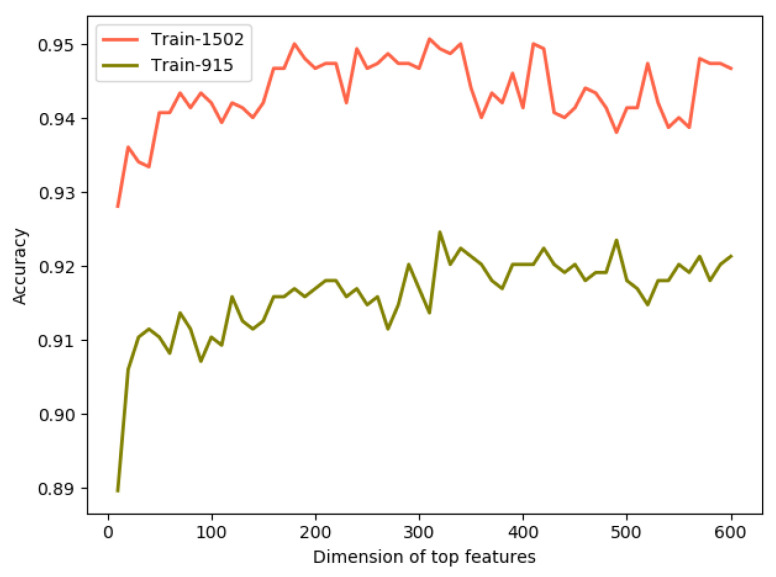
This graph illustrates how different top features affect the accuracies on the two datasets.

The two major benefits of feature selection are that it can enhance the generalization of a classifier by reducing overfitting and shorten the training time by avoiding the redundant and noisy features. In this section, we showed the effect of the feature selection algorithm on the performance of T4SEs identification. For this experiment, we performed the jackknife CV test on the two datasets to find the optimal sets of features by using the two-step feature selection method based on the RF algorithm. First, each protein sequence was encoded as a 3660-dimensional (3660D) feature vector and these features were ranked according to their Gini importance scores. Then, the top K features were input into the SVM classifier to select the most informative feature subset, where K=10, 20, 30, …, 600. Figure 4 illustrated the results on the Train-915 and Train-1502 datasets. As can be seen from Figure 4, for the Train-915 dataset, the highest Acc of 0.924 was obtained when K increased to 320. For the Train-1502 dataset, the Acc reached a maximum value of 0.950 when K was equal to 180.

Additionally, we compared the performance of the two models with and without feature selection on the two datasets, as shown in Table 2. For the Train-915 dataset, the model with feature selection outperformed the one based on the all features in terms of Acc (0.924), MCC (0.828), Spe (0.963), Pre (0.921), and F-score (0.882), which suggested that the performance of our predictor could be improved by eliminating the redundant features. For the Train-1502 dataset, the performance of the 180D-based model was slightly inferior to that of the model with all features. This demonstrated that the feature selection technique may reduce the computation time without incurring much loss of information. Therefore, two optimal models iT4SE-EP (320D) and iT4SE-EP (180D) were constructed on the Train-915 and Train-1502 datasets, respectively. Clearly, there remained the imbalance between the Sen and the Spe due to the uneven dataset. The Sen values could be increased appropriately by reducing the probability cut-off to 0.4 without much loss of the Spe. The detailed results were listed in Supplementary Table S2.

### 2.4. Peformance Comparision with Existing Methods

In this section, we further verified the robustness of the proposed model by performing two independent dataset tests. For a fair comparison with the state-of-the-art methods, the same training datasets and testing datasets were adopted. Accordingly, the iT4SE-EP (320D) model trained with the Train-915 dataset was examined on the Test-850 dataset, and the iT4SE-EP (180D) model trained with the Train-1502 dataset was evaluated on the Test-180 dataset, respectively. The prediction results of our method and several existing models were reported in Table 3 and Table 4.

As illustrated in Table 3, compared with Wang et al.’s method [15] and PredT4SE-Stack [7], iT4SE-EP (320D) obtained the best Acc, MCC, Spe, Pre, and F-score (0.956, 0.766, 0.962, 0.697, and 0.783, respectively) on the Test-850 dataset. In addition, the Sen of our predictor is 0.893, slightly lower than the highest value 0.907 of Wang et al.’s method [15]. It is worth mentioning that PredT4SE-Stack utilized the 400 PSSM-composition features to represent protein sequence samples and achieved the comparable performance with the help of a stacked ensemble classifier. This indicated that ensemble classifiers tended to yield better predictive results by aggregating multiple learning algorithms.

Table 4 listed the comparison results of iT4SE-EP (180D) and three annotation tools on the Test-180 dataset, including T4SEpre [4], Bastion4 [9], and CNN-T4SE [11] (the data were collected from the previous study [11]). In total, three T4SEpre classifiers (i.e., T4SEpre_psAac, T4SEpre_Joint, and T4SEpre_bpbAac), which only took account of C-terminal sequential and structural features, obtained relatively low MCC (from 0.537 to 0.620), Sen (from 0.367 to 0.500), and F-score (from 0.523 to 0.638). This may be due to the lack of the whole-length sequence information. To address this problem, Bastion4 improved the performance of T4SEs prediction with the Acc of 0.95 by extracting three main types of features such as local sequence encoding, global sequence encoding, and structural descriptor encoding. Besides, the CNN-PSSM, CNN-Onehot, and CNN-PSSSA models showed the powerful recognition capability of the deep learning algorithm for the annotation of T4SEs, with the Acc higher than 0.95, the F-score better than 0.85 and the MCC more than 0.8. In particular, CNN-PSSM obtained the overall best performance in terms of Acc, F-score, and MCC. It is also remarkable that our method gave the best Sen and the comparable Acc (0.966 vs. 0.989) and Spe (0.96 vs. 0.993) compared with CNN-PSSM when only 180D feature vectors were used.

In summary, the proposed iT4SE-EP model achieved the impressive performance and outperformed most of the existing tools for the prediction of T4SEs. We hope that our method could be effectively used for the large-scale annotation of T4SEs and at least play an important complementary role for the experimental validation of T4SEs.

## 3. Materials and Methods

### 3.1. Datasets

From the perspective of machine learning, the prerequisite step in developing an annotation tool of T4SEs is to establish a high-quality benchmark dataset for training and verifying the prediction model. In this study, 380 T4SEs and 1385 non-T4SEs were directly collected from a previous study to examine the performance of our proposed method [15]. These proteins in this dataset have mutual sequence similarity no more than 30%. Using the same strategy in that work [15], the total 1765 protein samples were divided into the training dataset (termed Train-915) and the independent test dataset (termed Test-850). Specifically, the training dataset consists of 305 T4SEs and 610 non-T4SEs, which were randomly selected from the positive and negative classes at a ratio of 1:2. Meanwhile, the remaining 75 T4SEs and 775 non-T4SEs were adopted as the independent test dataset.

To objectively evaluate the robustness of our predictor, another dataset constructed by Wang et al. [9] was further studied. This dataset also consists of two parts: the first one contains 390 T4SEs and 1112 non-T4SEs (termed Train-1502), which was used to train the model and perform the CV; and the other one includes 30 T4SEs and 150 non-T4SEs (termed Test-180), which was applied for the independent test.

### 3.2. Feature Extraction

#### 3.2.1. PSI-BLAST Profiles

A growing number of studies have found that evolution information encoded in the PSI-BLAST profiles could provide important clues for a wide variety of protein function classification tasks [18]. In this study, the PSSM and PSFM profiles of a query protein with the length of L are two L × 20 matrices, which were generated by performing the PSI-BLAST search against the UniRef50 [20] database with a threshold value of 0.001 and three iterations [19]. The (i, j)-th elements of two resulting profiles represent the log odds and weighted observed percentages of the j-th amino acid occurring at the i-th position of the query sequence during evolution, respectively. To facilitate the subsequent analysis, the elements of two matrices were normalized to reduce the noise and bias using the following formulas:

(1) $$f\left(x\right)=\frac{1}{{1+e}^{-x}}$$

(2) g(x)=x/100

The standardized PSSM and PSFM profiles are accordingly denoted as follows:

(3) $${P=[p}_{i,j}]\;\left(1\;\leq \;i\;\leq \;L,\;1\;\leq \;j\;\leq \;20\right)$$

(4) $${M=[m}_{i,j}]\;\left(1\;\leq \;i\;\leq \;L,\;1\;\leq \;j\;\leq \;20\right)$$

#### 3.2.2. Amino Acid Composition

AAC is represented as a 20-dimensional feature vector that calculates the frequencies of 20 standard amino acids in a protein sequence [21]. Given a PSSM, the corresponding AAC descriptor is defined as:

(5) $$\mathrm{PSSM}\_\mathrm{AAC}=\left[\bar{P_{1}},\bar{P_{2}},\ldots ,\bar{P_{20}}\right]$$

where $\bar{P_{j}}=\frac{1}{L}\sum_{i=1}^{L}p_{i,j}\left(1\;\leq \;j\;\leq \;20\right).$

#### 3.2.3. Evolutionary Difference Transformation

The EDT was often used to measure the non-co-occurrence probability of two amino acids with a position interval of d in a protein during evolution [22]. The resulting feature vector computed from the PSSM profile can be denoted as:

(6) $$\mathrm{PSSM}\_\mathrm{EDT}=\left[f_{x,y,d}\right]$$

where

(7) $$f_{x,y,d}=\frac{1}{L-d}\overset{L-d}{\underset{i=1}{\sum }}{\left(p_{i,x}-p_{i+d,y}\right)}^{2}\;\left(1\leq x,y\leq 20,\;1\leq d\leq D\right)$$

and D represents the maximum value of d. Hence, the size of the PSSM_EDT descriptor is 400 × D.

#### 3.2.4. Auto-Covariance and Cross-Covariance

The physicochemical properties of amino acids written into a protein sequence are commonly believed to be key determinants of the protein structure and function. Kidera et al. demonstrated that the 20 natural amino acids can be well represented by the 10 linearly independent property factors, which account for an 86% variance of the 188 selected properties [23]. Thus, a property factor matrix (PFM) can be applied to encode 20 amino acids, denoted by

(8) $${H=[h}_{i,j}]\;\left(1\;\leq \;i\;\leq \;20,\;1\;\leq \;j\;\leq 10\right)$$

where $h_{i,j}$ is the j-th property factor of the i-th amino acid.

Here we first employed PSFM and PFM to generate the position-specific physicochemical properties (PSPCP) using the similar strategy designed by Du and Yu [24]. The PSPCP is defined as a matrix of L × 10, which is derived from the product of two matrices M and H. Each element in the PSPCP is given by the following formula:

(9) $$s_{i,j}=\overset{20}{\underset{k=1}{\sum }}m_{i,k}h_{k,j}\;\left(1\;\leq \;i\leq \;L,\;1\;\leq \;j\;\leq \;10\right)$$

Then, we computed the ACC features from the PSPCP as described previously [25]:

(10) $$\mathrm{PSPCP}\_\mathrm{ACC}=\left[t_{x,y,g}\right]$$

where

(11) $$t_{x,y,g}=\frac{1}{L-g}\overset{L-g}{\underset{i=1}{\sum }}\left(s_{i,x}-\bar{S_{x}}\right)\left(s_{i+g,y}-\;\bar{S_{y}}\right)\;\left(1\;\leq \;x,y\;\leq \;10,\;1\;\leq \;g\;\leq \;G\right)$$

(12) $$\bar{S_{j}}=\frac{1}{L}\overset{L}{\underset{i=1}{\sum }}s_{i,j}\;\left(1\;\leq \;j\;\leq \;10\right)$$

and G is the maximum value of the lag g. By setting G=10 based on the past experience, we obtained a 1000-dimensional feature vector for the ACC descriptor.

#### 3.2.5. Discrete Wavelet Transform

The DWT has recently become very popular when it comes to analysis, de-noising and compression of signals. The outputs of a single-level 1-dimensional DWT are composed of two parts: the approximation coefficients vector and the detail coefficients vector. The former represents the high-scale and low-frequency components of input signals, while the latter is opposite [26].

In this study, each column of a PSFM was first treated as a set of the discrete signal. Next, inspired by Nanni’s work [27], the biorthogonal 3.3 wavelet type was chosen with a decomposition level of 4 to perform the 1-dimensional DWT. Finally, we computed the first five discrete cosine values from the approximation coefficients, and the mean, minimum, maximum, and standard deviation values of both the approximation and detail coefficients. As a result, the 1040 features were extracted as the PSFM_DWT descriptor.

### 3.3. Feature Normalization

In machine learning, the trained model will not work properly without the normalization of features because the range of original features varies widely. Therefore, feature scaling is generally considered to be an important preprocessing step towards improving the performance of the predictive models. In this study, the min–max normalization was adopted to rescale the raw features into the range of 0 to 1, given by

(13) $$X^{\prime }=\frac{X-\min\left(X\right)}{\max\left(X\right)-\min\left(X\right)}$$

where X is the original value, and $X^{\prime }$ is the normalized value.

### 3.4. Model Construction

#### 3.4.1. Support Vector Machine

As one of the most powerful supervised learning algorithms, SVM has been successfully applied to an increasingly wide variety of bioinformatics applications, especially the protein classification tasks [28,29,30]. Theoretically, SVM maps the training examples to points in a high-dimensional space so as to find the maximum-margin hyperplane which might separate the two categories. New testing examples are then mapped into the same space and predicted to belong to a category based on which side of the hyperplane they fall. In addition to handling linearly separable datasets, SVM performs well on the non-linear classification problems by using the kernel trick. The common kernels include linear, polynomial, radial basis function (RBF), and sigmoid [31]. In this study, the RBF kernel was adopted to carry out the prediction because it often gives better results in the previous studies. The two parameters C and γ were optimized in the ranges of $\left\{2^{-5}{,2}^{-3}{,\ldots ,2}^{13}{,2}^{15}\right\}$ and $\left\{2^{3}{,2}^{5}{,\ldots ,2}^{-13}{,2}^{-15}\right\}$ by running a grid search program.

#### 3.4.2. Random Forest

As the name suggests, RF is a tree-based ensemble predictor that fits a number of decision tree classifiers on various sub-samples of the given dataset and takes averaging to improve the predictive accuracy. Actually, RF makes full use of two popular machine learning techniques: bagging and random selection of features. In bagging, each tree is trained on a random sample with replacement of the training set, and predictions are made by taking the majority vote of trees. RF is similar to bagging except that a random subset of the features is selected at each candidate split when growing a tree [32]. Since the RF algorithm is simple in theory and easy to realize, it has a wide range of applications in bioinformatics [33,34].

Unlike most other classifiers, RF can directly perform feature selection by calculating the importance of each feature while classification rules are built. Feature importance score is used to rank these features and then the optimal feature subset can be selected by means of an incremental stepwise greedy method. Both Gini importance index and permutation importance index are the two commonly used variable important measures based on RF [35]. In this study, the former was adopted and the RF algorithm was implemented by utilizing the scikit-learn library in Python [36].

### 3.5. Performance Evaluation

To impartially measure the performance of the proposed method, we carried out two types of validation tests: the jackknife CV and the independent dataset test, accompanied by the following six quantitative metrics. They include sensitivity (Sen), specificity (Spe), accuracy (Acc), F-score, precision (Pre), and Matthew’s correlation coefficient (MCC), defined as follows:

(14) $$\mathrm{Sen}=\frac{\mathrm{TP}}{\mathrm{TP}+\mathrm{FN}}$$

(15) $$\mathrm{Spe}=\frac{\mathrm{TN}}{\mathrm{TN}+\mathrm{FP}}$$

(16) $$\mathrm{Acc}=\frac{\mathrm{TP}+\mathrm{TN}}{\mathrm{TP}+\mathrm{FP}+\mathrm{TN}+\mathrm{FN}}$$

(17) $$\text{F-score}=2\;\times \;\frac{\mathrm{TP}}{2\mathrm{TP}+\mathrm{FP}+\mathrm{FN}}$$

(18) $$\mathrm{Pre}=\frac{\mathrm{TP}}{\mathrm{TP}+\mathrm{FP}}$$

(19) $$\mathrm{MCC}=\frac{\left(\mathrm{TP}\;\times \;\mathrm{TN}\right)\;-\;\left(\mathrm{FN}\;\times \;\mathrm{FP}\right)}{\sqrt{\left(\mathrm{TP}+\mathrm{FN}\right)\;\times \;\left(\mathrm{TN}+\mathrm{FP}\right)\;\times \;\left(\mathrm{TP}+\mathrm{FP}\right)\;\times \;\left(\mathrm{TN}+\mathrm{FN}\right)}}$$

where TP, FP, TN, and FN represent the numbers of the true positive, false positive, true negative, and false negative, respectively. In addition, the receiver operating characteristic (ROC) curve was created to visually illustrate the diagnostic ability of our predictor by plotting the true positive rate (TPR) against the false positive rate (FPR) at different thresholds. Note that TPR is equivalent to Sen, and FPR is equal to 1-Spe. The area under the ROC curve (AUC) was also computed and provided in the ROC figure, as a reliable performance measure.

## 4. Conclusions

The biological significance of effector proteins has motivated the development of computational tools that facilitate accurate annotation of T4SEs based on their protein sequences alone. In this study, we proposed a method iT4SE-EP to further improve the predictive performance of T4SEs by exploring the evolutionary information encoded in two PSI-BLAST profiles. Firstly, four types of feature descriptors were designed to represent all the T4SEs and non-T4SEs from the working datasets, including PSSM_AAC, PSPCP_ACC, PSFM_DWT, and PSSM_EDT. Secondly, the RF algorithm was implemented to rank all features according to their Gini importance scores and then the optimal 320 and 180 features were selected to construct the final SVM-based predictors, respectively. Thirdly, both the jackknife CV and the independent test were performed to verify the performance of iT4SE-EP. Comparison with the state-of-the-art predictors demonstrated that our method exhibited the impressive improvement and could serve as a useful tool for identifying T4SEs. All the datasets and the source codes for this study are freely available at https://github.com/taigangliu/iT4SE-EP. In the future, we will develop a user-friendly web server for public use.

## Figures and Tables

**Table 1:** Prediction results of the SVM and RF classifiers with different features.

Method	Feature	Acc	Sen	Spe	Pre	F-Score	MCC	AUC
SVM	PSSM_AAC (20D)	0.908	0.836	0.944	0.882	0.858	0.791	0.928
	PSPCP_ACC (1000D)	0.834	0.645	0.929	0.820	0.722	0.616	0.868
	PSFM_DWT (1040D)	0.902	0.793	0.957	0.902	0.844	0.777	0.927
	PSSM_EDT (1600D)	0.920	0.845	0.957	0.908	0.876	0.818	0.943
	All features (3660D)	0.919	0.845	0.955	0.905	0.874	0.816	0.935
RF	PSSM_AAC (20D)	0.904	0.809	0.952	0.894	0.850	0.782	0.933
	PSPCP_ACC (1000D)	0.803	0.563	0.922	0.785	0.656	0.537	0.834
	PSFM_DWT (1040D)	0.893	0.777	0.952	0.890	0.830	0.757	0.924
	PSSM_EDT (1600D)	0.900	0.806	0.947	0.884	0.843	0.772	0.940
	All features (3660D)	0.907	0.816	0.952	0.895	0.854	0.788	0.937

Note: The values in parentheses represent the dimension of the feature vector.

**Table 2:** Performance comparison before and after feature selection on the two datasets.

Dataset	Feature	Acc	Sen	Spe	Pre	F-Score	MCC
Train-915	All features (3660D)	0.919	0.845	0.955	0.905	0.874	0.816
	Optimal subset (320D)	0.924	0.845	0.963	0.921	0.882	0.828
Train-1502	All features (3660D)	0.954	0.879	0.981	0.942	0.909	0.880
	Optimal subset (180D)	0.950	0.861	0.981	0.941	0.899	0.867

**Table 3:** Performance comparison on the Test-850 dataset.

Method	Acc	Sen	Spe	Pre	F-Score	MCC
Wang et al.’s method [15]	0.853	0.907	0.848	0.366	0.521	0.518
PredT4SE-Stack (SVM)	0.945	0.867	0.952	0.637	0.734	0.715
PredT4SE-Stack (LR)	0.944	0.880	0.950	0.629	0.733	0.715
iT4SE-EP (320D)	0.956	0.893	0.962	0.697	0.783	0.766

**Table 4:** Performance comparison on the Test-180 dataset.

Method	Acc	Sen	Spe	Pre	F-Score	MCC
T4SEpre_psAac	0.889	0.367	0.993	0.917	0.523	0.537
T4SEpre_Joint	0.906	0.500	0.987	0.882	0.638	0.620
T4SEpre_bpbAac	0.889	0.433	0.980	0.813	0.565	0.541
Bastion4	0.950	0.967	0.947	0.784	0.865	0.842
CNN-PSSSA	0.956	0.767	0.993	0.958	0.851	0.833
CNN-Onehot	0.967	0.800	1.000	1.000	0.888	0.877
CNN-PSSM	0.989	0.967	0.993	0.967	0.966	0.960
iT4SE-EP (180D)	0.966	1.000	0.96	0.833	0.909	0.894

## Data Availability

The data used to support the findings of this study are freely available to the academic community at https://github.com/taigangliu/iT4SE-EP.

## Supplementary Materials

The following are available online, Table S1: Prediction results of the SVM and RF classifiers based on different features with the cut-off of 0.4, Table S2: Performance comparison before and after feature selection with the cut-off of 0.4.

## Sample Availability

Samples of the compounds are not available from the authors.

## References

[1] Desvaux M., Hebraud M., Talon R., Henderson I.R. (2009). Secretion and subcellular localizations of bacterial proteins: A semantic awareness issue. Trends Microbiol..

[2] Bi D., Liu L., Tai C., Deng Z., Rajakumar K., Ou H.-Y. (2013). SecReT4: A web-based bacterial type IV secretion system resource. Nucleic Acids Res..

[3] Meyer D.F., Noroy C., Moumene A., Raffaele S., Albina E., Vachiery N. (2013). Searching algorithm for type IV secretion system effectors 1.0: A tool for predicting type IV effectors and exploring their genomic context. Nucleic Acids Res..

[4] Wang Y., Wei X., Bao H., Liu S.-L. (2014). Prediction of bacterial type IV secreted effectors by C-terminal features. BMC Genom..

[5] Noroy C., Lefrancois T., Meyer D.F. (2019). Searching algorithm for Type IV effector proteins (S4TE) 2.0: Improved tools for Type IV effector prediction, analysis and comparison in proteobacteria. PLoS Comput. Biol..

[6] Zou L., Nan C., Hu F. (2013). Accurate prediction of bacterial type IV secreted effectors using amino acid composition and PSSM profiles. Bioinformatics.

[7] Xiong Y., Wang Q., Yang J., Zhu X., Weil D.-Q. (2018). PredT4SE-Stack: Prediction of Bacterial Type IV Secreted Effectors From Protein Sequences Using a Stacked Ensemble Method. Front. Microbiol..

[8] Xue L., Tang B., Chen W., Luo J. (2018). A deep learning framework for sequence-based bacteria type IV secreted effectors prediction. Chemom. Intell. Lab. Syst..

[9] Wang J., Yang B., An Y., Marquez-Lago T., Leier A., Wilksch J., Hong Q., Zhang Y., Hayashida M., Akutsu T. (2019). Systematic analysis and prediction of type IV secreted effector proteins by machine learning approaches. Brief. Bioinform..

[10] Ashari Z.E., Brayton K.A., Broschat S.L. (2019). Prediction of T4SS Effector Proteins for Anaplasma phagocytophilum Using OPT4e, A New Software Tool. Front. Microbiol..

[11] Hong J., Luo Y., Mou M., Fu J., Zhang Y., Xue W., Xie T., Tao L., Lou Y., Zhu F. (2020). Convolutional neural network-based annotation of bacterial type IV secretion system effectors with enhanced accuracy and reduced false discovery. Brief. Bioinform..

[12] Chen T., Wang X., Chu Y., Wang Y., Jiang M., Wei D.-Q., Xiong Y. (2020). T4SE-XGB: Interpretable Sequence-Based Prediction of Type IV Secreted Effectors Using eXtreme Gradient Boosting Algorithm. Front. Microbiol..

[13] Burstein D., Zusman T., Degtyar E., Viner R., Segal G., Pupko T. (2009). Genome-Scale Identification of Legionella pneumophila Effectors Using a Machine Learning Approach. PLoS Pathog..

[14] An Y., Wang J., Li C., Leier A., Marquez-Lago T., Wilksch J., Zhang Y., Webb G.I., Song J., Lithgow T. (2018). Comprehensive assessment and performance improvement of effector protein predictors for bacterial secretion systems III, IV and VI. Brief. Bioinform..

[15] Wang Y., Guo Y., Pu X., Li M. (2017). Effective prediction of bacterial type IV secreted effectors by combined features of both C-termini and N-termini. J. Comput.-Aided Mol. Des..

[16] Zhavoronkov A., Ivanenkov Y.A., Aliper A., Veselov M.S., Aladinskiy V.A., Aladinskaya A.V., Terentiev V.A., Polykovskiy D.A., Kuznetsov M.D., Asadulaev A. (2019). Deep learning enables rapid identification of potent DDR1 kinase inhibitors. Nat. Biotechnol..

[17] Eraslan G., Avsec Z., Gagneur J., Theis F.J. (2019). Deep learning: New computational modelling techniques for genomics. Nat. Rev. Genet..

[18] Wang J., Yang B., Revote J., Leier A., Marquez-Lago T.T., Webb G., Song J., Chou K.-C., Lithgow T. (2017). POSSUM: A bioinformatics toolkit for generating numerical sequence feature descriptors based on PSSM profiles. Bioinformatics.

[19] Altschul S.F., Madden T.L., Schaffer A.A., Zhang J.H., Zhang Z., Miller W., Lipman D.J. (1997). Gapped BLAST and PSI-BLAST: A new generation of protein database search programs. Nucleic Acids Res..

[20] Suzek B.E., Wang Y., Huang H., McGarvey P.B., Wu C.H., UniProt C. (2015). UniRef clusters: A comprehensive and scalable alternative for improving sequence similarity searches. Bioinformatics.

[21] Liu T., Zheng X., Wang J. (2010). Prediction of protein structural class for low-similarity sequences using support vector machine and PSI-BLAST profile. Biochimie.

[22] Zhang L., Zhao X., Kong L. (2014). Predict protein structural class for low-similarity sequences by evolutionary difference information into the general form of Chou’s pseudo amino acid composition. J. Theor. Biol..

[23] Kidera A., Konishi Y., Oka M., Ooi T., Scheraga H.A. (1985). Statistical analysis of the physical properties of the 20 naturally occurring amino acids. J. Protein Chem..

[24] Du P., Yu Y. (2013). SubMito-PSPCP: Predicting Protein Submitochondrial Locations by Hybridizing Positional Specific Physicochemical Properties with Pseudoamino Acid Compositions. Biomed Res. Int..

[25] Dong Q., Zhou S., Guan J. (2009). A new taxonomy-based protein fold recognition approach based on autocross-covariance transformation. Bioinformatics.

[26] Shensa M.J. (1992). The discrete wavelet transform: Wedding the a trous and Mallat algorithms. IEEE Trans. Signal Process..

[27] Nanni L., Brahnam S., Lumini A. (2012). Wavelet images and Chou’s pseudo amino acid composition for protein classification. Amino Acids.

[28] Noble W.S. (2006). What is a support vector machine?. Nat. Biotechnol..

[29] Bressin A., Schulte-Sasse R., Figini D., Urdaneta E.C., Beckmann B.M., Marsico A. (2019). TriPepSVM: De novo prediction of RNA-binding proteins based on short amino acid motifs. Nucleic Acids Res..

[30] Garg A., Singhal N., Kumar R., Kumar M. (2020). mRNALoc: A novel machine-learning based in-silico tool to predict mRNA subcellular localization. Nucleic Acids Res..

[31] Cortes C., Vapnik V. (1995). Support-vector networks. Mach. Learn..

[32] Breiman L. (2001). Random forests. Mach. Learn..

[33] Jiang P., Wu H., Wang W., Ma W., Sun X., Lu Z. (2007). MiPred: Classification of real and pseudo microRNA precursors using random forest prediction model with combined features. Nucleic Acids Res..

[34] Hooghe B., Broos S., van Roy F., De Bleser P. (2012). A flexible integrative approach based on random forest improves prediction of transcription factor binding sites. Nucleic Acids Res..

[35] Qi Y., Zhang C., Ma Y. (2012). Random Forest for Bioinformatics. Ensemble Machine Learning: Methods and Applications.

[36] Pedregosa F., Varoquaux G., Gramfort A., Michel V., Thirion B., Grisel O., Blondel M., Prettenhofer P., Weiss R., Dubourg V. (2011). Scikit-learn: Machine Learning in Python. J. Mach. Learn. Res..

