# Supplementary material for: iT4SE-EP: Accurate Identification of Bacterial Type IV Secreted Effectors by Exploring Evolutionary Features from Two PSI-BLAST Profiles
Source: Molecules. 2021 Apr 24;26(9):2487. doi: 10.3390/molecules26092487 (PMC8123216; doi:10.3390/molecules26092487)
Supplement: Supplementary file 1 [file molecules-26-02487-s001.zip › molecules-1167213-supplementary.pdf]

Table S1. Prediction results of the SVM and RF classifiers based on different features with the cut-off of 0.4.

Method	Feature	Acc	Sen	Spe	Pre	F-score	MCC	AUC
SVM	PSSM_AAC (20D)	0.907	0.849	0.936	0.869	0.859	0.789	0.927
	PSPCP_ACC (1000D)	0.827	0.714	0.883	0.754	0.734	0.606	0.868
	PSFM_DWT (1040D)	0.904	0.849	0.932	0.863	0.856	0.785	0.927
	PSSM_EDT (1600D)	0.916	0.852	0.949	0.893	0.872	0.811	0.942
	All features (3660D)	0.914	0.865	0.939	0.877	0.871	0.807	0.935
RF	PSSM_AAC (20D)	0.901	0.845	0.929	0.857	0.851	0.777	0.932
	PSPCP_ACC (1000D)	0.781	0.695	0.824	0.664	0.679	0.514	0.833
	PSFM_DWT (1040D)	0.895	0.849	0.918	0.838	0.843	0.764	0.923
	PSSM_EDT (1600D)	0.902	0.852	0.927	0.855	0.853	0.780	0.940
	All features (3660D)	0.900	0.845	0.927	0.854	0.850	0.775	0.936

Table S2. Performance comparison before and after feature selection with the cut-off of 0.4.

Dataset	Feature	Acc	Sen	Spe	Pre	F-score	MCC
Train-915	All features (3660D)	0.914	0.865	0.939	0.877	0.871	0.807
	Optimal subset (320D)	0.918	0.852	0.950	0.896	0.873	0.813
Train-1502	All features (3660D)	0.953	0.892	0.974	0.925	0.908	0.877
	Optimal subset (180D)	0.946	0.874	0.971	0.914	0.893	0.858
